# The “Now” and “Next” in Precision Cardiovascular Medicine

**DOI:** 10.3390/biomedicines13112697

**Published:** 2025-11-03

**Authors:** Panteleimon Pantelidis, Polychronis E. Dilaveris

**Affiliations:** School of Medicine, National & Kapodistrian University of Athens, 115 27 Athens, Greece

## 1. A State-of-the-Art and Forward Look at Personalized Cardiovascular Care

The convergence of Artificial Intelligence (AI), genomic medicine, and precision therapeutics is fundamentally transforming cardiovascular care from reactive treatment paradigms toward proactive, individualized approaches. This Special Issue, “Cardiovascular Diseases in the Era of Precision Medicine”, presents comprehensive works that collectively illuminate how technological innovation is reshaping our understanding, diagnosis, and management of cardiovascular disease. These contributions span the breadth of precision medicine. From Artificial Intelligence-enhanced diagnostics to genetic modifier discovery, and from vascular imaging innovations to novel therapeutic strategies, each advances our capacity to deliver truly personalized cardiovascular care.

## 2. Artificial Intelligence: Unlocking Hidden Disease Patterns

As a starter, this Issue hosts a comprehensive exploration of AI’s role in cardiovascular medicine through the review “Hearts, Data, and Artificial Intelligence Wizardry: From Imitation to Innovation in Cardiovascular Care”. This work transcends conventional perspectives on AI as merely an automation tool, instead articulating a vision where AI tackles problems fundamentally beyond human cognitive capacity. The authors demonstrate that AI’s true strength lies not in replicating human decision-making but in integrating multimodal data streams—electrocardiography, echocardiography, cardiac magnetic resonance imaging, genomics, and wearable sensor data—to uncover latent disease patterns that are typically imperceptible through traditional methods [1]. The evidence presented is compelling: AI models can achieve impressive performances when tackling human-intractable problems, such as detecting atrial fibrillation during sinus rhythm (accuracy > 85%), and predicting heart failure from standard electrocardiograms months before symptom onset. These capabilities represent a paradigm shift from feature-based diagnosis to data-driven discovery of novel disease markers. The authors emphasize AI’s consistency in eliminating cognitive fatigue and bias while enabling continuous real-time monitoring. Similarly, AI demonstrates a capacity to distinguish genetic cardiomyopathy subtypes through electrocardiographic and echocardiographic patterns, moving cardiovascular diagnostics toward more “proactive” risk stratification [2,3].

However, the authors appropriately acknowledge persistent challenges: algorithmic opacity, potential biases in training datasets, and generalization difficulties with rare presentations. These limitations underscore the necessity for AI to complement rather than replace clinical expertise, augmenting human judgment in ways that extend diagnostic precision beyond conventional boundaries [1].

## 3. Expanding Diagnostic Horizons: Venous Disease and AI

The diagnostic applications of AI extend to even more diverse fields, including phlebological diseases, as explained in the review “Does Artificial Intelligence Bring New Insights in Diagnosing Phlebological Diseases?”. This comprehensive analysis of nine studies involving over 1000 patients reveals AI algorithms consistently achieving accuracies exceeding 90% across venous pathology detection tasks, including chronic venous disease, venous reflux, and deep venous thrombosis [4]. The clinical implications are substantial: AI reduces inter-observer variability while accelerating diagnostic workflows by more than 50%. Crucially, these systems excel at identifying subtle abnormalities (minor venous reflux and early-stage thrombi) which are frequently overlooked during manual evaluation.

The authors envision AI algorithms embedded directly into ultrasound devices, providing instantaneous diagnostic support during patient examinations and combining multimodal imaging approaches for comprehensive vascular assessment. This work establishes foundational evidence for AI-driven preventive strategies in venous disease management, transforming what has traditionally been a reactive field into a predictive discipline. The successful application of AI across imaging modalities, from Doppler ultrasound to thermal imaging, demonstrates the technology’s adaptability and potential for widespread clinical integration and serves as a paradigm [4].

## 4. Genomic Architecture: From Risk Prediction to Therapeutic Targeting

A comprehensive synthesis of genomic and precision medicine advances in atherosclerotic cardiovascular disease, entitled “Genomic and Precision Medicine Approaches in Atherosclerotic Cardiovascular Disease: From Risk Prediction to Therapy”, is also hosted in this Special Issue. This work masterfully integrates contemporary understanding of genetic variants, polygenic risk scores, and therapeutic applications derived from genomic discoveries [5]. The authors highlight transformative developments in PCSK9 research, where genetic discoveries have directly translated into precision therapeutics. PCSK9 inhibitors demonstrate 50–60% LDL cholesterol reductions and 27% decreases in myocardial infarction risk. This exemplifies precision medicine’s trajectory: understanding individual genetic variations guides targeted therapy development, moving from population-based interventions toward individualized treatment strategies.

Additionally, polygenic risk scores emerge as powerful tools for early risk stratification. In UK Biobank participants, individuals with high polygenic risk scores showed 72% increased coronary artery disease risk, 2.1-fold elevation in coronary artery calcium scores, and 26% greater plaque burden compared to low-risk individuals. When integrated with traditional clinical factors, polygenic risk scores significantly enhance predictive accuracy, enabling early identification of high-risk individuals who benefit from intensified preventive measures. The authors also explore cutting-edge developments in gene-editing technologies. CRISPR-Cas9 applications in non-human primate models have achieved an 83% reduction in circulating PCSK9 protein and a 69% decrease in LDL cholesterol with effects sustained over one year following single intravenous infusion. These findings support the possibility of long-term cholesterol control through single-treatment interventions, representing a quantum leap beyond daily medication regimens. Similarly, targeting ANGPTL3 through gene editing significantly reduces triglycerides and cholesterol levels, highlighting therapeutic potential for broader dyslipidemias [5,6].

## 5. Genetic Complexity: Modifier Genes in Arrhythmogenic Disease

Another bright work (“A Novel Bradycardia-Associated Variant in HCN4 as a Candidate Modifier in Type 3 Long QT Syndrome”) illuminates the complexity of genetic interactions in arrhythmogenesis. This three-generational family study demonstrates how secondary genetic variants significantly influence disease phenotype, adding crucial layers of understanding to apparently straightforward genetic diagnoses [7]. The discovery of a novel HCN4 variant (p.V642M) alongside the established SCN5A mutation (p.E1784K) provides compelling evidence for genetic modifier effects in long QT syndrome type 3. Through a sophisticated in silico analysis, the authors establish the causal role of the HCN4 variant in sinus bradycardia while suggesting its contribution to phenotypic heterogeneity among affected family members. The HCN4 p.V642M variant emerges as damaging, with evolutionary conservation analyses and structural predictions supporting its functional significance.

This work exemplifies precision medicine’s future, where understanding genetic interactions enables more accurate risk stratification and personalized management strategies. The presence of severe bradycardia in the proband’s sister, who is genotype-negative for long QT syndrome but harbors the HCN4 p.V642M variant, suggests this variant’s causal role in cardiac rhythm disturbances. The authors appropriately acknowledge the complexity of genotype–phenotype correlations, noting that sudden cardiac death occurred in a family member without the HCN4 variant, highlighting the multifactorial nature of clinical outcomes in inherited arrhythmias [7].

## 6. Novel Therapeutic Paradigms: Beyond Anticoagulation in Atrial Fibrillation

Another critical clinical challenge is addressed through the comprehensive review “Non-Anticoagulation Strategies Aimed at Primary Stroke Prevention in Nascent Atrial Fibrillation”: Patients with early-stage atrial fibrillation where traditional anticoagulation strategies may be inappropriate or insufficient due to low CHA_2_DS_2_-VA scores, uncertain embolic–hemorrhagic risk ratios, or difficulty obtaining electrocardiographic confirmation [8]. The authors systematically evaluate evidence-based alternatives including statins, antidiabetic medications, angiotensin pathway modulators, anti-inflammatory agents, and folate cycle fortification, with each targeting different pathophysiological mechanisms underlying stroke risk. Their systematic approach categorizes interventions by cardiovascular risk levels, providing practical guidance for clinicians managing complex patient populations. Of particular interest is the evidence for colchicine, which demonstrated a 28% stroke risk reduction in stable coronary artery disease patients (HR 0.72, 95% CI 0.57–0.92) in the LoDoCo-2 trial. Similarly, GLP-1 receptor agonists show significant stroke-preventive effects in type 2 diabetes patients, with meta-analyses demonstrating a 17% risk reduction (HR 0.83, 95% CI 0.76–0.92).

The review demonstrates how precision medicine extends beyond genetic testing to encompass comprehensive risk factor modification and targeted therapeutic interventions based on individual patient profiles and underlying pathophysiology. This approach acknowledges that atrial fibrillation-related stroke risk stems from multiple mechanisms beyond atrial hypercoagulability, including inflammation, endothelial dysfunction, and metabolic disturbances [8].

## 7. Future Directions: The Convergence Accelerates

The collective insights from these five studies illuminate several transformative trends shaping cardiovascular medicine’s future. The integration of AI with genomic data promises to revolutionize drug discovery while enabling precise therapeutic targeting. Foundation models (analogous to large language models but trained on cardiovascular data) will likely integrate genomic information with multimodal clinical data streams to provide real-time risk assessment, treatment optimization, and outcome prediction [9,10]. Digital twins, virtual representations of individual patients, are emerging as powerful tools for simulating treatment responses and optimizing therapeutic strategies without subjecting patients to potential risks. These computational models integrate physiological, anatomical, and molecular data to create dynamic patient replicas capable of predicting disease progression and treatment outcomes [11].

Gene-editing technologies are moving rapidly from laboratory to clinic, offering permanent genetic modifications addressing cardiovascular disease at molecular roots. Beyond the PCSK9 trials discussed in this issue, CRISPR-Cas9 has demonstrated remarkable success in correcting familial cardiomyopathy mutations. The ABEmax-VRQR-SpCas9 system successfully corrected RBM20 mutations in dilated cardiomyopathy models, resulting in reversal of cardiac dilation and restoration of normal cardiac function. In hypertrophic cardiomyopathy, single AAV9 delivery targeting the MYH6 R403Q mutation rendered the pathogenic allele inactive in over 70% of ventricular myocytes, effectively preventing disease hallmarks. Notably, CRISPR activation (CRISPRa) has achieved first-in-kind success treating cardiac arrhythmias in mice by restoring FLNC gene function, normalizing electrocardiographic abnormalities and completely eliminating drug-induced arrhythmias. These advances herald a future where genetic cardiovascular diseases may not only be treated but potentially cured through precision genome editing [6,12]. The future of precision medicine lies in integrating multi-omics approaches—genomics, transcriptomics, proteomics, epigenomics, metabolomics, and microbiomics—to offer a holistic understanding of cardiovascular disease pathogenesis. These combined data layers enable dynamic disease profiling, moving beyond static risk factors toward precise, individualized assessments that account for each patient’s unique biological signature. Network medicine approaches analyze molecular interaction networks to uncover previously unrecognized disease phenotypes, relationships between diseases, and potential drug interactions [9,13].

## 8. Challenges and Implementation Considerations

Despite remarkable progress, significant challenges persist. Data heterogeneity, algorithmic bias, and the need for diverse representation in training datasets pose ongoing concerns. The “black box” nature of many AI systems necessitates advances in explainable AI, such as saliency maps that highlight the areas of model input that drive a decision [14], in order to build clinical trust and enable meaningful interpretation of recommendations. Regulatory frameworks are evolving to address AI validation and ensure equitable access to advanced technologies. The integration of multi-omics data requires sophisticated computational infrastructure and analytical expertise that may not be readily available across all healthcare settings. Cost-effectiveness analyses and physician education programs will be crucial for widespread implementation of precision medicine approaches. Ethical considerations surrounding genetic data privacy, insurance discrimination, and germline editing require robust regulatory frameworks.

## 9. Conclusions: Toward Truly Personalized Cardiovascular Care

The articles in this Special Issue represent significant strides toward a future where every cardiovascular patient receives care tailored to their unique biological signature. From AI systems predicting heart failure before symptoms appear to genetic analysis revealing complex modifier interactions, and from innovative vascular imaging to novel therapeutic strategies beyond anticoagulation, these advances demonstrate medicine’s transformation from reactive treatment to proactive risk management. The convergence of AI, advanced genomics, digital health technologies, and innovative therapeutic strategies is creating unprecedented opportunities for predictive, preventive, personalized, and participatory medicine. Foundation models integrating genomic data with clinical information promise to accelerate drug discovery and enable precise therapeutic targeting. Gene therapies are expanding beyond rare diseases to address common cardiovascular conditions. Digital therapeutics and remote monitoring offer new modalities for delivering personalized interventions [2,6,9].

As we advance toward this vision of truly personalized cardiovascular care, the research presented here provides essential foundations for a healthcare system that genuinely understands, predicts, and prevents cardiovascular disease at the individual level. The future promises not merely incremental improvements but a fundamental reimagining of cardiovascular care: moving from population-based protocols to individualized strategies honoring the complexity and uniqueness of human biology. The integration of AI-enhanced diagnostics, genome-informed therapeutics, digital twin simulations, and precision pharmacology will enable clinicians to deliver care optimized for each patient’s genetic makeup, environmental exposures, lifestyle factors, and molecular phenotype. The future of cardiovascular care is personalized, predictive, and profoundly promising.

## Data Availability

Not applicable.

## Abbreviations

The following abbreviations are used in this manuscript:

AAV9	Adeno-Associated Virus serotype 9
ABEmax-VRQR-SpCas9	Advanced gene-editing system generally explained as an Optimized Adenine Base Editor (ABEmax) that utilizes a variant (VRQR) of the Streptococcus pyogenes Cas9 (SpCas9) enzyme
AI	Artificial Intelligence
ANGPTL3	Angiopoietin Like 3 (A protein involved in lipid metabolism)
CHA_2_DS_2_-VA	Risk stratification score: Congestive heart failure, Hypertension, Age ≥75 years, Diabetes mellitus, Stroke/TIA/Thromboembolism, Vascular disease, Age 65–74 years
CI	Confidence Interval
CRISPR	Clustered Regularly Interspaced Short Palindromic Repeats
FLNC	Filamin C
GLP-1	Glucagon-Like Peptide-1
HCN4	Hyperpolarization Activated Cyclic Nucleotide Gated Potassium Channel 4
HR	Hazard Ratio
LDL	Low-Density Lipoprotein
LoDoCo-2	Low-Dose Colchicine for Secondary Prevention of Cardiovascular Disease 2
MYH6	Myosin Heavy Chain 6
PCSK9	Proprotein convertase subtilisin/kexin type 9
RBM20	RNA Binding Motif Protein 20
SCN5A	Sodium Voltage-Gated Channel Alpha Subunit 5
UK	United Kingdom
